# Risk factors for non-participation in ivermectin and dihydroartemisinin-piperaquine mass drug administration for malaria control in the MASSIV trial

**DOI:** 10.1186/s12936-024-04878-2

**Published:** 2024-02-22

**Authors:** Christian Kositz, Hristina Vasileva, Nuredin Mohammed, Jane Achan, Edgard Diniba Dabira, Umberto D’Alessandro, John Bradley, Michael Marks

**Affiliations:** 1 Clinical Research Department, Faculty of Infectious and Tropical Diseases, London School of Hygiene & Tropical Medicine, Keppel Street, London, UK; 2 Disease Control and Elimination, Medical Research Council Unit Gambia at the London School of Hygiene and Tropical Medicine (MRCG at LSHTM), Fajara, The Gambia; 3 Department of Infectious Disease Epidemiology, Faculty of Epidemiology and Population Health, London School of Hygiene & Tropical Medicine, Keppel Street, London, UK; 4 Hospital for Tropical Diseases, University College London Hospital, London, UK; 5 Division of Infection and Immunity, University College London, London, UK

**Keywords:** MDA, Non-compliance, Ivermectin

## Abstract

**Background:**

Mass Drug Administration (MDA) has become a mainstay for the control of several diseases over the last two decades. Successful implementation of MDA programmes requires community participation and can be threatened by systematic non-participation. Such concerns are particularly pertinent for MDA programmes against malaria, as they require multi-day treatment over several consecutive months. Factors associated with non-participation to the MDA campaign with ivermectin (IVM) and dihydroartemisinin-piperaquine (DHP) implemented within the MASSIV cluster randomized trial were determined.

**Methods:**

Coverage data was extracted from the MASSIV trial study database, with every datapoint being a directly observed therapy (DOT). A complete month of MDA was classified as receiving all three daily doses of treatment. For both ivermectin and DHP, ordinal logistic regression was used to identify individual and household level variables associated with non-participation.

**Results:**

For ivermectin, 51.5% of eligible participants received all 3 months of treatment while 30.7% received either one or two complete months. For DHP, 56.7% of eligible participants received all 3 months of treatment and 30.5% received either one or two complete months. Children aged 5–15 years and adults aged more than 50 years were more likely to receive at least one complete month of MDA than working age adults, both for ivermectin (aOR 4.3, 95% CI 3.51–5.28 and aOR of 2.26, 95% CI 1.75–2.95) and DHP (aOR 2.47, 95%CI 2.02–3.02 and aOR 1.33, 95%CI 1.01–1.35), respectively.

Members of households where the head received a complete month of MDA were more likely to themselves have received a complete month of MDA, both for ivermectin (aOR 1.71, 95%CI 1.35–2.14) and for DHP (aOR 1.64, 95%CI 1.33–2.04).

**Conclusion:**

Personal and household-level variables were associated with participation in the MDA programme for malaria control. Specific strategies to (increase participation amongst some groups may be important to ensure maximum impact of MDA strategies in achieving malaria elimination.

*Trial registration*: The MASSIV trial is registered under NCT03576313.

**Supplementary Information:**

The online version contains supplementary material available at 10.1186/s12936-024-04878-2.

## Background

Mass drug administration (MDA) has become established as a mainstay in the control of various diseases over the past 15–20 years [1, 2]. Successful MDA programmes require cooperation with local communities to ensure high coverage of the treated population. For many MDA-based interventions, the degree to which individuals participate in MDA programmes over a number of years is an important consideration. The impact of MDA may be markedly reduced by systematic non-compliance rather than random non-compliance of participants or households, as missing specific segments of the population may have a particularly large impact [3–7].

Previous studies have suggested that non-participation in MDA programmes does not occur at random. Instead, systematic non-participation is often observed and may be linked to both individual and household-level factors such as the participation of the household head [8, 9]. The impact of systematic non-participation likely varies depending on the extent to which MDA is providing direct treatment of individuals or indirect effects on transmission of a pathogen, or a mix of both. When the primary effect is on transmission, high levels of coverage can still result in a significant impact on the disease, even if a section of the population is systematically excluded.

Ivermectin is among the most commonly used drugs for MDA programmes and is currently in programmatic use for the control of several neglected tropical diseases (NTDs) [10]. More recently, the drug has been identified to have the potential to become a malaria vector control tool, due to its mosquitocidal effects on *Anopheles* spp. when taken with a blood meal [11]. In contrast to NTD programmes that administer a single annual dose of ivermectin, to achieve significant mosquitocidal effects, ivermectin has to be taken at a higher dose for multiple consecutive days. Taking 300–400 mcg/kg bodyweight of ivermectin for 3 days in a row generates an effect on mosquito populations for up to 28 days and is safe and well tolerated [12]. However, repeated ivermectin dosing, requiring both multiple consecutive days and multiple months of treatment, may cause challenges in maintaining high population coverage which could ultimately impact the efficacy of this intervention.

The MASSIV trial conducted in the Upper River Region in the eastern part of The Gambia is the first large scale trial to investigate the effects of MDA with ivermectin (IVM) and dihydroartemisinin-piperaquine (DHP), an anti-malarial treatment, in an integrated MDA, with both drugs being given on three consecutive days for three consecutive months. Given the pharmacokinetic characteristics of ivermectin, the success of these MDA programmes is likely to be highly dependent on the coverage of the intervention. The trial therefore provides the ideal opportunity to investigate the frequency and determinants of systematic non-compliance participation in more complex MDA programmes.

In this study, data from the MASSIV trial was used to assess both individual and household- level factors that influence uptake and coverage of the trial intervention.

## Methods

### Study design

The MASSIV (NCT03576313) trial design, baseline findings, and outcomes have been described previously [13, 14]. In summary, 32 clusters (villages) were randomized (1:1) to either the intervention or the control arm. The intervention clusters received MDA orally with IVM at a dose of 300–400 mcg/kg bodyweight plus DHP at 320/40 mg and 160/20 mg depending on body weight on three consecutive days at monthly intervals for 3 months during at the start into the malaria season (July–October), which coincides with the geographical wet season. The MDA schedule is based on modelling data that suggests ivermectin would exert its mosquitocidal effect for up to 28 days at this dose when taken on three consecutive days [12].

Exclusion criteria for IVM were chronic illnesses, bodyweight under 15 kg measured at distribution, pregnancy, excluded with point of care pregnancy tests, and hypersensitivity to the drug. Exclusion criteria for DHP were < 6 months of age, first trimester pregnancy, hypersensitivity to the drug, and cardiac QTc prolonging medication. The intervention was carried out in 2018 and 2019. The primary outcome was malaria prevalence measured by qPCR after MDA and the results of this have been previously reported [14]. A tertiary outcome was soil-transmitted helminth (STH) and prevalence of ectoparasites, which were thought to be specifically affected by ivermectin (rather than DHP) and has been reported elsewhere [15].

### Study setting and population

The trial was implemented in Upper River Region (URR), eastern Gambia. The population included several ethnic groups, specifically, Fula, Mandinka, Sarahule, and Wollof. Malaria transmission occurs during the rainy season (June-November) and the following 2 months. The database for this study included every registered participant in the 16 villages in URR included in the intervention arm. As part of the study the MASSIV trial staff conducted community sensitization, including involvement of villages’ health workers, birth attendants, women & youth organization in mobilization of the villages’ residents and social events during the MDA within the communities. Study participants were visited each day of treatment, which was directly observed (three consecutive days on each of three consecutive months). At baseline, data was collected on demographics including age and gender, the household size and the head of the household. On each study day eligible participants were offered ivermectin and DHP (as outlined above). For each study day, the team recorded if either, both, one, or no drugs were taken by each participant. No drugs were left with the family if a member was absent. In these circumstances, the field teams attempted to recontact absent family members later in the day when farming activities had ceased.

### Statistical analysis

Patterns of compliance were analysed in the intervention arm of the 2nd year of the MASSIV trial. The main outcome was the number of months in which a participant received all three doses; this ranged from 0 to 3 completed months of MDA, with 1 month equating to one course of MDA and a dose equating 1 daily dose. The analysis was done separately for IVM and DHP. In addition, as the dosing required for an impact on NTDs differs from that for malaria, receiving no MDA or at least one dose of MDA was looked at.

The proportion of individuals who received 0–3 completed monthly courses of ivermectin and DHP was calculated separately for each drug in-line with their specific inclusion and exclusion criteria. Frequencies of non-treatment within and across rounds were displayed with histograms generated with the UpSetR package in R software with R studio version 4.0.2 (2020-06-22) [16].

For the primary outcome of a complete monthly course of MDA, an ordered logistic regression model that included fixed effects for age group, sex, household size, ethnicity, and treatment status of the household head, and random effects for study clusters (villages) was used. Age was stratified into five groups, < 5, 5–15, > 15–25, > 25–50 and over > 50 years of age with the 25–50 years age group used as reference. The median household size of 25 members per household was used to stratify the study population into five groups, < 6, 6–12, > 12–24, 25–50 and > 50 members per household. Data on ethnicity was self-reported and included members of Fula, Mandinka, Sarahule, Wollof, and not specified. Participation of household heads was categorised in complete, partial or no MDA received. For the analysis of receiving of at least one dose of ivermectin a logistic regression model was fitted using a similar approach.

In a post hoc analysis, interactions between the treatment status for household heads with age group (< 5, 5–15 and > 15 years of age) and with sex was assessed. For these analyses the outcome variable was a complete monthly course of MDA. Statistical analysis was conducted using STATA 17.

### Ethics statement

Ethical clearance for the MASSIV study was provided by the ethics board of the London School of Hygiene and Tropical Medicine, and the Gambian government/MRCG Joint Ethics Committee (Ethics Ref. Nr. 15,823).

## Results

**Fig. 1:**
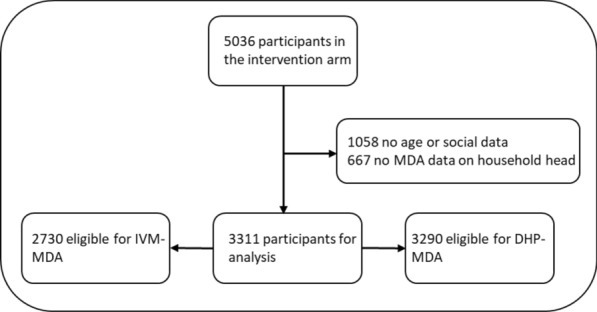
Number of participants in the intervention arm and their exclusion criteria for this study. *IVM*  ivermectin, *DHP*  dihydroartemisinin-piperaquine

The MASSIV database contained 5036 participants in the intervention arm of which 3311 had complete data. Within this population, 2730 were eligible for ivermectin MDA and 3291 for DHP-MDA. 1058 people were excluded due to missing age or social data and 667 due to no available data on the MDA status of the household head (Fig. 1). Most participants (n = 2065, 62.4%) were Fula, followed by Mandinka (638, 19.3%), Sarahule (134, 4.1%), Wollof (3, 0.1%); 471 (14.2%) did not specify their ethnicity. There were more females than males (Table 1). Data on the MDA coverage of individuals with missing demographic data is reported in Additional file 1: Table S1.

**Table 1:** Baseline demographics of the intervention group of the MASSIV database

Number of participants selected for analysis from the intervention Arm (%)	
Total	3311 (100.0)
Age	
< 5 years	585 (17.7)
5–15 years	1065 (32.2)
> 15–25 years	543 (16.4)
> 25–50	780 (23.6)
> 50 years	338 (10.2)
Sex	
Female	1780 (53.8)
Male	1531 (46.2)
Ethnicity	
Fula	2065 (62.4)
Mandinka	638 (19.3)
Sarahule	134 (4.1)
Wollof	3 (0.1)
Not specified	471 (14.2)
Household Size	
< 6	51 (1.5)
6–11	391 (11.8)
12–24	1373 (41.5)
25–50	1141 (34.5)
> 50	355 (10.7)
MDA Eligible	
DHP	3291 (99.4)
Ivermectin	2730 (82.5)

### Ivermectin MDA

**Fig. 2:**
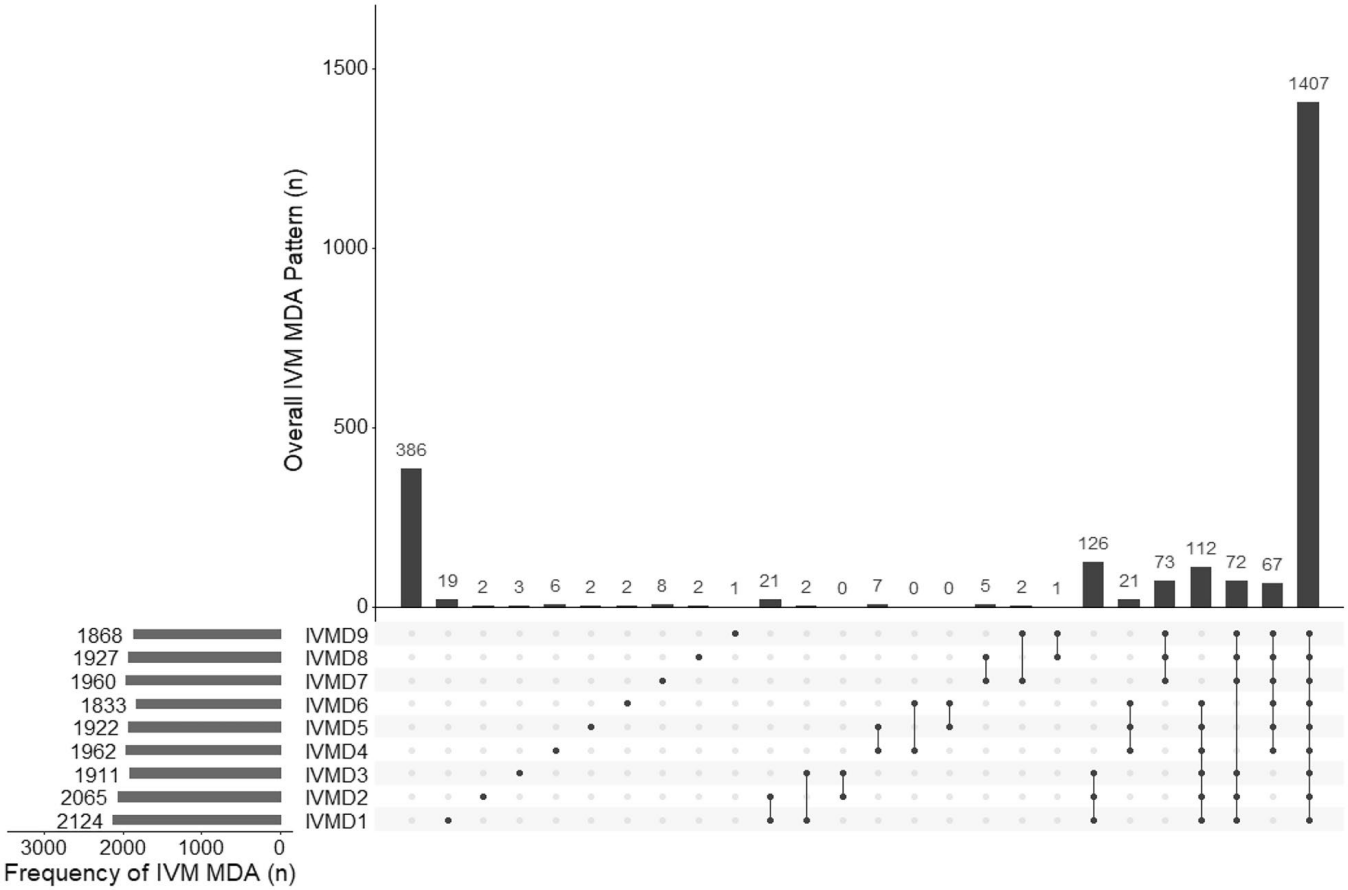
Overall treatment frequency and pattern for the ivermectin MDA excluding non-eligible participants. Full points indicate MDA received. IVMD# denotes the number of ivermectin doses received, and the pattern such as a single dose or all nine MDA rounds. Certain patterns, such as received 1st and 9th MDA but none in between are excluded for convenience, therefore data for only 2347/2730 participants are show are shown

Excluding non-eligible participants, 1407/2730 (51.5%) participants received all three-monthly courses of ivermectin MDA, 327 (11.9%) received only one complete monthly course, 512 (18.8%) received only two complete monthly courses and 484 (12.8%) did not receive any MDA or did not complete a single monthly round of MDA (Table 2, Fig. 2, Additional file 1: Figs. S1 and S2).

**Table 2:** Ivermectin treatment by demographic factors

	Total Population (N = 3311)				
	Eligible population/total population (%)	Eligible population for ivermectin (N = 2370)			
		No MDA complete (%)	1 month of MDA complete (%)	2 months of MDA complete (%)	3 months of MDA complete (%)
Total	2730/3311 (82.5)	484 (17.73)	327 (11.98)	512 (18.8)	1407 (51.5)
Houshold head MDA					
Complete	1445/1754 (82.4)	222 (15.3)	140 (9.7)	242 (16.8)	841 (58.2)
Incomplete	816/1006 (81.1)	149 (18.2)	131 (16.1)	186 (22.8)	350 (42.9)
None	469/551 (85.1)	113 (24.1)	56 (11.9)	84 (17.9)	216 (46.1)
Age group					
< 5 years	116/585 (19.8)	10 (8.6)	16 (13.8)	31 (26.7)	59 (50.9)
5–15 years	1010/1065 (94.8)	35 (3.5)	70 (6.9)	177 (17.5)	728 (72.1)
>15–25 years	524/543 (96.5)	157 (30)	84 (16)	110 (21)	173 (33)
>25–50	742/780 (95.1)	233 (31.4)	110 (14.8)	129 (17.4)	270 (36.4)
> 50 years	338/338 (100)	49 (14.5)	47 (13.9)	65 (19.2)	177 (52.4)
Sex					
Female	1457/1780 (81.9)	309 (21.2)	183 (12.6)	253 (17.4)	712 (48.9)
Male	1273/1531 (83.1)	175 (13.8)	144 (11.31)	259 (20.4)	695 (54.6)
Ethnicity					
Fula	1716/2065 (83.1)	166 (9.7)	206 (12)	353 (20.6)	991 (57.8)
Mandinka	526/638 (82.4)	54 (10.3)	44 (8.4)	89 (16.9)	339 (64.5)
Sarahule	115/134 (85.8)	8 (6.9)	9 (7.8)	23 (20)	75 (65.2)
Wollof	3/3 (100)	0 (0)	0 (0)	1 (33.3)	2 (66.7)
Not specified	370/471 (78.6)	256 (69.2)	68 (18.4)	46 (12.4)	0 (0)
Household size					
< 6	46/51 (90.2)	5 (10.9)	5 (10.9)	11 (23.9)	25 (54.4)
6–11	322/391 (82.4)	57 (17.7)	55 (17.1)	61 (18.9)	149 (46.3)
12–24	1137/1373 (82.8)	195 (17.2)	119 (10.5)	195 (17.2)	628 (55.2)
25–50	937/1141 (82.1)	168 (17.9)	104 (11.1)	177 (18.9)	488 (52.1)
> 50	288/355 (81.1)	59 (20.5)	44 (15.3)	68 (23.6)	117 (40.6)

Eligible children under 5 with an aOR of 1.43 (95%CI 0.97–2.1), children aged 5–15 years with an aOR of 4.3 (95%CI 3.51–5.28) and older adults > 50 years of age with an aOR of2.27 (95%CI 1.75–2.95) were all more likely to receive MDA than adolescents and young working age adults aged 15–25, and 25–50 years. Males were also more likely than women to receive at least one complete month of MDA with an aOR of 1.54 (95%CI 1.31–1.81). Neither household size nor ethnicity were associated with receiving a complete month of ivermectin MDA, except individuals who did not specify their ethnicity, who had an aOR of 0.05 (95%CI 0.04–0.06) for receiving one complete month of MDA (Table 3). Individuals living in a house where the household head received a complete course of MDA were more likely to receive a complete month of MDA with an aOR of 1.71 (95%CI 1.35–2.14) than those living in households whose head did not receive a complete course of MDA (Table 3).

**Table 3:** aORs from ordered logistic regression for participants eligible for ivermectin receiving a completed month of MDA

Variables associated with receiving ivermectin amongst the eligible population (by completed Nr of monthly MDAs)				
	aOR	95% CI	p—value for specific variables	Likelihood Ratio—p—value
Household head receiving MDA				
None	1			< 0.001
Complete	1.71	1.35–2.14	< 0.001	
Incomplete	1.14	0.89–1.47	0.287	
Household size				
< 6	1			0.0457
> 6–12	0.64	0.33–1.25	0.198	
> 12–25	0.75	0.39–1.42	0.379	
> 25–50	0.59	0.31–1.14	0.119	
> 50	0.53	0.26–1.05	0.069	
Ethnicity				
Fula	1			< 0.001
Mandinka	1.04	0.74–1.45	0.8	
Sarahule	1.16	0.64–2.11	0.61	
Wollof	1.14	0.11–11.5	0.911	
Not specified	0.05	0.04–0.06	< 0.001	
Age group				
< 5	1.43	0.97–2.10	0.065	< 0.001
> 5–15	4.3	3.51–5.28	< 0.001	
> 15–25	0.92	0.73–1.14	0.45	
> 25–50	1			
> 50	2.27	1.75–2.95	< 0.001	
Sex				
Female	1			< 0.001
Male	1.54	1.31–1.81	< 0.001	

There was no evidence of an interaction between an individual’s age and whether the household heads received MDA with an interaction p-value of 0.171 (Additional file 1: Table S2). In contrast, there was strong evidence of an interaction with the gender of adult participants, with adult males much more likely to receive MDA compared to women if the household head had received MDA with an interaction p-value of 0.0001 (Additional file 1: Table S3).

For the secondary analysis of receiving at least a single dose of ivermectin, eligible children aged under 5 with an aOR of 4.61 (95%CI 1.52–13.97), children aged 5–15 (8.81 95%CI 5.51–14.07) and older adults aged > 50 years with an aOR of 3.43 (95%CI 2.13–5.48) were more likely to receive treatment. Men were also more likely than women to receive at least one dose of IVM with an aOR of 2.71 (95%CI 1.98–3.71). Neither household size nor ethnicity appeared to be associated with receiving at least one dose of IVM (Additional file 1: Table S4).

### DHP MDA

**Fig. 3:**
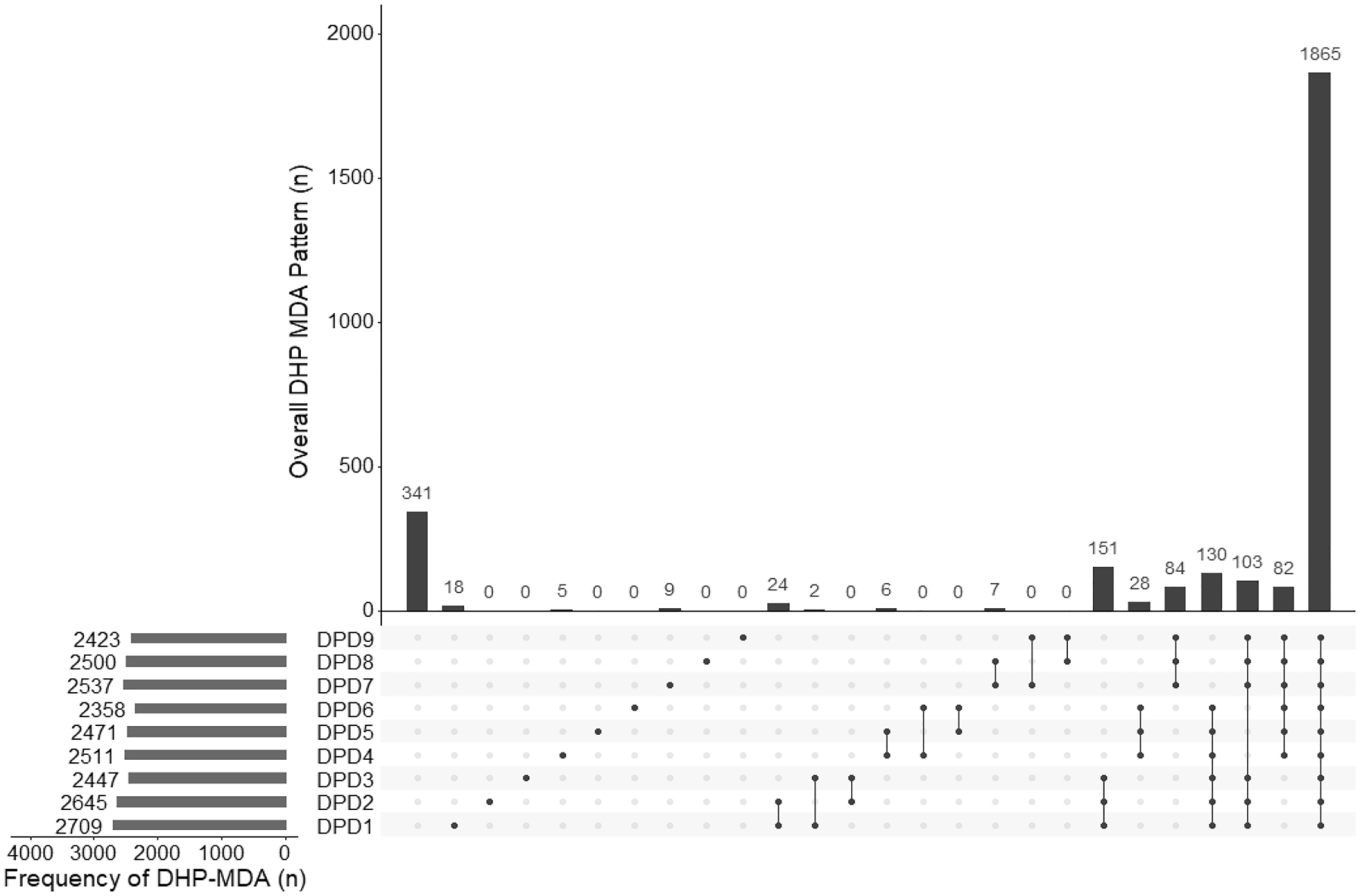
Overall treatment frequency and pattern for the dihydroartemisinin-piperaquine (DHP) MDA excluding non-eligible participants. Full points indicate MDA received. DPD# denotes the number of doses of DHP received, and the pattern such as a single dose or all nine MDA rounds. Certain patterns, such as received 1st and 9th MDA but none in between are excluded for convenience, therefore data for only 2855/3290 participants are shown

Excluding non-eligible participants, 1865/3290 (56.7%) participants received all 3 months of DHP MDA, 623/3290 (18.9%) received only two complete months of MDA. 380/3290 (11.6%) received only one complete month of MDA and 422 (12.8%) did not receive any complete months of DHP MDA (Table 4, Fig. 3 and Additional file 1: Figs. S1 and S2).

**Table 4:** DHP treatment by demographic factors

	Total population (N = 3311)				
	Eligible population/total population (%)	Eligible Population for DHP (N = 3290)			
		No MDA complete (%)	1 month of MDA complete (%)	2 months of MDA complete (%)	3 months of MDA complete (%)
Total	3290/3311 (99.4)	422 (12.8)	380 (11.6)	623 (18.9)	1865 (56.7)
Houshold Head MDA					
Complete	1742/1754 (99.3)	191 (10.9)	162 (9.3)	281 (16.1)	1108 (63.6)
Incomplete	999/1006 (99.3)	128 (12.8)	155 (15.5)	240 (24)	476 (47.7)
None	549/551 (99.6)	103 (18.8)	63 (11.4)	102 (18.6)	281 (51.2)
Agegroup					
< 5 years	564/585 (96.4)	47 (8.3)	51 (9)	104 (18.4)	362 (64.2)
5–15 years	1065/1065 (100)	65 (6.1)	69 (6.5)	167 (15.7)	764 (71.7)
> 15– 25 years	543/543 (100)	117 (21.6)	92 (16.9)	127 (23.4)	207 (38.1)
> 25–50	780/780 (100)	145 (18.6)	121 (15.5)	159 (20.4)	355 (45.5)
> 50 years	338/338 (100)	48 (14.2)	47 (13.9)	66 (19.5)	177 (52.4)
Sex					
Female	1768/1780 (99.3)	230 (13)	225 (12.7)	332 (18.8)	981 (55.5)
Male	1522/1531 (99.4)	192 (12.6)	155 (10.2)	291 (19.1)	884 (58.1)
Ethnicity					
Fula	2062/2065 (99.9)	88 (4.3)	228 (11.1)	425 (20.6)	1321 (64.1)
Mandinka	637/638 (99.8)	30 (4.7)	49 (7.7)	108 (16.9)	450 (70.7)
Sarahule	134/134 (100)	6 (4.5)	12 (8.9)	25 (18.7)	91 (67.9)
Wollof	3/3 (100)	0 (0)	0 (0)	0 (0)	3 (100)
Not specified	454/471 (96.4)	298 (65.6)	91 (20)	65 (14.3)	0 (0)
Household size					
< 6	51/51 (100)	6 (11.8)	5 (9.8)	11 (21.6)	29 (56.8)
6–11	390/391 (99.7)	48 (12.3)	60 (15.4)	75 (19.2)	207 (53)
12–24	1365/1373 (99.4)	163 (11.9)	144 (10.6)	236 (17.3)	822 (60.2)
25–50	1136/1141 (99.5)	149 (13.1)	121 (10.7)	219 (19.3)	647 (56.9)
> 50	348/355 (98)	56 (16.1)	50 (14.4)	82 (23.6)	160 (45.9)

Similar to our findings for IVM MDA, eligible children under 5 with an aOR of 2.01 (95%CI 1.59–2.54), children aged 5–15 with an aOR or 2.47 (95%CI 2.02–3.02) and older adults > 50 years with an aOR of 1.33 (95%CI 1.01–1.35) were all more likely to receive at least one complete month of DHP MDA than working age adults. Males were also more likely than females to receive at least one complete month of MDA with an aOR of 1.17 (95%CI: 1.01.–1.35). Similar to IVM MDA, neither household size nor ethnicity were associated with receiving a complete month of DHP MDA. Individuals living in a house where the household head received a complete month of MDA were more likely to receive a complete month of MDA themselves with an aOR of 1.64 (95%CI 1.33–2.04) than those living in households whose head did not receive any complete months of treatment (Table 5).

**Table 5:** aORs from ordered logistic regression for participants eligible for DHP receiving a completed month of MDA

Variables associated with receiving DHP amongst the eligible population (by completed Nr of monthly MDAs)				
	aOR	95% CI	p—value for specific variables	Likelihood ratio p—value
Household head receiving MDA				
None	1			< 0.001
Complete	1.64	1.33–2.04	< 0.001	
Incomplete	1.07	0.85–1.34	0.564	
Household size				
< 6	1			0.06
> 6–12	0.85	0.45–1.62	0.628	
> 12–25	0.94	0.51–1.75	0.858	
> 25–50	0.77	0.41–1.43	0.41	
> 50	0.66	0.34–1.28	0.219	
Ethnicity				
Fula	1			< 0.001
Mandinka	1.05	0.76–1.46	0.74	
Sarahule	0.78	0.44–1.39	0.399	
Wollof	6.21E + 07	(0 -.)	0.998	
Not specified	0.03	0.02–0.03	0.021	
Age group				
< 5	2.01	1.59–2.54	< 0.001	< 0.001
> 5–15	2.47	2.02–3.02	< 0.001	
> 15–25	0.76	0.61–0.94	0.014	
> 25–50	1			
> 50	1.33	1.01–1.35	0.036	
Sex				
Female	1			0.036
Male	1.17	1.01–1.35	0.036	

There was evidence of a weak interaction between age groups and the impact of whether a household head had received MDA (interaction p-value of 0.039). Similar to the findings for IVM MDA, strong evidence of an interaction between the gender of adults and the MDA status of the household head, the males were much more likely to receive MDA if the household head had also been treated (interaction p-value < 0.0005) (Additional file 1: Tables S2 and S3).

## Discussion

Within the MASSIV trial, some individuals and households did not participate across multiple monthly rounds of an integrated ivermectin and DHP MDA programme delivered for malaria control. Systemic non-compliance has been previously recognized as a threat to the successful implementation of MDA interventions [4, 9, 17]. This may be particularly true for ivermectin when used for malaria vector control in addition with or without an additional drug, as the MDA schedule requires several doses of MDA per month over 3 months in a relatively short timeframe and a relatively high coverage to result in a measurable impact [18, 19].

The MASSIV trial provided a unique opportunity to explore the factors associated with non-participation in more detail and used the data to inform future ivermectin-DHP MDA rollouts in other settings. Similar factors were associated with MDA participation for ivermectin and DHP, suggesting that individuals either participated in all or no parts of the intervention.

In keeping with previous studies, there was a strong relationship between participation of the household head and the other members of the family. Similar associations with household head participation have been reported from The Gambia in the context of MDA with azithromycin for trachoma elimination [8]. These data highlight the key role family structures have on the participation in some community-based health interventions such as MDA. Notably the lowest rate of participation was seen in adolescents, young adults and working aged individuals who are often absent during public health interventions. Previous studies on MDA for soil-transmitted helminths and onchocerciasis [3] have also found high rates of absenteeism in these groups [20]. Collectively, data across these studies highlight the fact that specific measures such as adjusting the timing of MDA delivery may be required to increase uptake of MDA amongst these population segments.

In line with other studies, a strong association was found in regards to gender and receiving MDA, with men being more likely than women to receive both ivermectin and DHP [20, 21]. It might be anticipated that exclusion criteria related to pregnancy or breastfeeding would affect the uptake of ivermectin, however a similar phenomenon would not be expected for DHP. Previous studies have highlighted that not receiving ivermectin during previous pregnancies can encourage women not to participate to the MDA even if not pregnant [20, 21], alternatively some women may have not wanted to complete a pregnancy test due to a lack of privacy [22]. Men have also been reported to be more likely to receive MDA for soil transmitted helminths in Kenya but no difference between sexes was seen amongst children in a previous study examining participation in trachoma MDA in the Gambia or in adults for soil-transmitted helminths in Uganda [3, 6, 23]. Continuous monitoring is required to facilitate equal distribution of treatment during MDA and ensure women are not more likely to be excluded from such interventions.

The major limitation of our study was that we had to exclude a fifth of the participations from the analysis due to lack of complete demographic data. However, accounting for the fact that a proportion of excluded participants likely had an age < 5, the overall distribution of MDA participation was similar to the individuals whose data was available (see Additional file 1: Table S1). In addition, there was no information on socioeconomic factors such as household income, education levels and road access, some of which have previously been reported to be associated with the likelihood of participating in MDA interventions [7, 24] nor whether non-participation reflected absence at the time of MDA or declining to take treatment. Finally, only data from the 2nd year of the MASSIV trial was used due to difficulties with the data collection in the 1st year. It would be important to assess if similar evidence of systematic non-participation was observed over a multi-year MDA cycle.

This study highlights several key areas that must be addressed to optimize the use of ivermectin as a potential tool for malaria vector control. Engagement with household heads must be a central pillar of such strategies as their participation influences the entire household. In particular, enhanced strategies to improve coverage amongst adolescents and working age adults should be considered such as amending or adapting the MDA timing, considering evening drug distribution and potentially improved engagement with the community in the implementation of the intervention. The data shows that MDA implementation must be adapted to the participating community, its cultural background, infrastructural realities on the ground, such as agricultural seasons in particular. Addressing these findings will be key to achieve the maximum benefit of ivermectin MDA for malaria control.

### Supplementary Information

**Additional file 1: Table S1.** MDA coverage of individuals missing demographic data. **Table S2.** Interaction between age-groups and MDA status of household heads. **Table S3.** Interaction between sex and MDA status of household heads. **Table S4.** Factors associated with receiving at least one dose of ivermectin. **Figure S1.** Overall number of doses received for both DHP and IVM MDA for eligible participants. **Figure S2.** Overall number of doses received for both drugs for eligible participants including missing demographic data.

## Data Availability

After publication, trial data will be made available on request to the corresponding author. A proposal with a detailed description of the study objectives and a statistical analysis plan is needed for the evaluation of the requests. Additional materials might also be required during the process.

## Abbreviations

DHP: Dihydroartemisinin-piperaquine
DPD: Dihydroartemisinin-piperaquine doses
DOT: Directly observed therapy
IVM: Ivermectin
MASSIV: Mass Drug Administration of Ivermectin and Dihydroartemisinin-piperaquine as an Additional Intervention for Malaria Elimination
MDA: Mass Drug Administration
NTD: Neglected Tropical Diseases
qPCR: Quantitative polymerase chain reaction
STH: Soil-transmitted helminths
URR: Upper River Region
