# Supplementary material for: Risk factors for non-participation in ivermectin and dihydroartemisinin-piperaquine mass drug administration for malaria control in the MASSIV trial
Source: Malar J. 2024 Feb 22;23:54. doi: 10.1186/s12936-024-04878-2 (PMC10882911; doi:10.1186/s12936-024-04878-2)
Supplement: Supplementary file 1 — Additional file 1: Table S1. MDA coverage of individuals missing demographic data. Table S2. Interaction between age-groups and MDA status of household heads. Table S3. Interaction between sex and MDA status of household heads. Table S4. Factors associated with receiving at least one dose of ivermectin. Figure S1. Overall number of doses received for both DHP and IVM MDA for eligible participants. Figure S2. Overall number of doses received for both drugs for eligible participants including missing demographic data. [file 12936_2024_4878_MOESM1_ESM.docx]

**Additional Files**

**Supplementary Table 1**. MDA coverage of individuals missing demographic data

**Supplementary Table 2.** Interaction between age-groups and MDA status of household heads

**Supplementary Table 3.** Interaction between sex and MDA status of household heads

**Supplementary Table 4**. Factors associated with receiving at least one dose of ivermectin

**Supplementary Figure 1.** Overall number of doses received for both DHP and IVM MDA for eligible participants

**Supplementary Figure 2.** Overall number of doses received for both drugs for eligible participants including missing demographic data

**Supplementary Tables**

**Supplementary Table 1.** MDA coverage of individuals missing demographic data

| **Characteristic** |  | **No Month (%)** | **1 Month MDA (%)** | **2 Months MDA (%)** | **3 Months MDA (%)** |
| --- | --- | --- | --- | --- | --- |
| **Population Missing Data (DHP MDA)** | 1,726 (100.0) | 242 (14.0) | 311 (18.0) | 377 (21.8) | 796 (46.1) |
|  |  |  |  |  |  |
| **Population Missing Data (IVM)** | 1,726 (100.0) | 543 (31.5)* | 265 (15.4) | 303 (17.6) | 615 (35.6) |

Table shows the distribution of the excluded participants who either lacked age, social data or household head data; *lack of age data might lead to inclusion of <5-year-olds who are not eligible for IVM MDA

**Supplementary Table 2.** Interaction between age groups and MDA status of household heads

|  |  | Ivermectin | | DHP | |
| --- | --- | --- | --- | --- | --- |
| Age | Head of house hold | OR (95%CI) | p – value | OR | p - value |
| < 5 | None | 1 | 0.75 | 1 | 0.72 |
|  | Complete | 0.74 (0.08 - 6.77) |  | 1.05 (0.41 - 2.7) |  |
|  | Incomplete | 0.48 (0.05 - 4.70) |  | 0.81 (0.31 - 2.13) |  |
| 5 - 15 | None | 1 | 0.82 | 1 | 0.96 |
|  | Complete | 0.86 (0.33 - 2.20) |  | 0.99 (0.5 - 1.96) |  |
|  | Incomplete | 1.09 (0.376 - 3.18) |  | 0.93 (0.44 - 1.94) |  |
| > 15 | None | 1 | < 0.001 | 1 | < 0.001 |
|  | Complete | 2.23 (1.62 - 3.04) |  | 2.38 (1.7 - 3.35) |  |
|  | Incomplete | 1.74 (1.24 - 2.45) |  | 1.99 (1.37 - 2.89) |  |
| Interaction p-value | | 0.171 | | 0.039 | |

**Supplementary Table 3.** Interaction between sex and MDA status of household heads

|  |  | Ivermectin | | DHP | |
| --- | --- | --- | --- | --- | --- |
| Age > 15 | Head of Household | OR | p-value | OR | p - value |
| Sex |  |  |  |  |  |
| Female | None | 1 | 0.3 | 1 | 0.357 |
|  | Complete | 1.35 (0.9 - 2.02) |  | 1.37 (0.85 - 2.2) |  |
|  | Incomplete | 1.15 (0.73 - 1.81) |  | 1.44 (0.84 - 2.45) |  |
| Male | None | 1 | < 0.001 | 1 | < 0.001 |
|  | Complete | 4.75 (2.86 - 7.91) |  | 4.75 (2.86 - 7.91) |  |
|  | Incomplete | 2.85 (1.67 - 4.85) |  | 2.93 (1.72 - 5.00) |  |
| Interaction p-value | | 0.0001 |  | 0.0005 |  |

**Supplementary Table 4.** Factors associated with receiving at least one dose of ivermectin

| Variables associated with receiving ivermectin amongst the eligible population (IVM No/Yes) (%) | | | | | |
| --- | --- | --- | --- | --- | --- |
|  |  | aOR | 95% CI | p - value for specific variables | Likelihood Ratio p - value |
| Household Size | | | | | |
| <6 | 43/46 (93.5) | 1 |  |  | 0.175 |
| >6 - 12 | 272/322 (84.5) | 0.23 | 0.05 - 1.09 | 0.065 |  |
| >12 - 25 | 986/1137 (86.7) | 0.24 | 0.056 - 1.08 | 0.064 |  |
| >25 - 50 | 800/937 (85.4) | 0.2 | 0.044 - 0.89 | 0.035 |  |
| > 50 | 243/288 (84.4) | 0.25 | 0.05 - 1.18 | 0.082 |  |
|  |  |  |  |  |  |
| Ethnicity | | | | | |
| Fula | 1607/1716 (93.7) | 1 |  |  | 0.84 |
| Mandinka | 495/526 (94.1) | 1.37 | 0.74 - 2.53 | 0.304 |  |
| Sarahule | 108/115 (93.9) | 1.48 | 0.51 - 4.38 | 0.47 |  |
| Wollof | 3/3 (100) | 1 | - | - |  |
| Not specified | 131/370 | 0.032 | 0.02 - 0.04 | < 0.001 | < 0.001 |
|  |  |  |  |  |  |
| Age Group | | | | | |
| <5 | 112/116 (96.6) | 4.61 | 1.52 - 13.97 | 0.007 | < 0.001 |
| >5 - 15 | 983/1010 (97.3) | 8.81 | 5.51 - 14.07 | < 0.001 |  |
| >15 - 25 | 397/524 (75.8) | 1.15 | 0.83 - 1.61 | 0.83 |  |
| >25 - 50 | 552/742 (74.4) | 1 |  |  |  |
| >50 | 300/338 (88.8) | 3.43 | 2.13 - 5.48 | < 0.001 |  |
|  |  |  |  |  |  |
| Sex | | | | | |
| Female | 1209/1457 (82.9) | 1 |  |  | < 0.001 |
| Male | 1135/1273 (89.2) | 2.71 | 1.98 - 3.71 | < 0.001 |  |


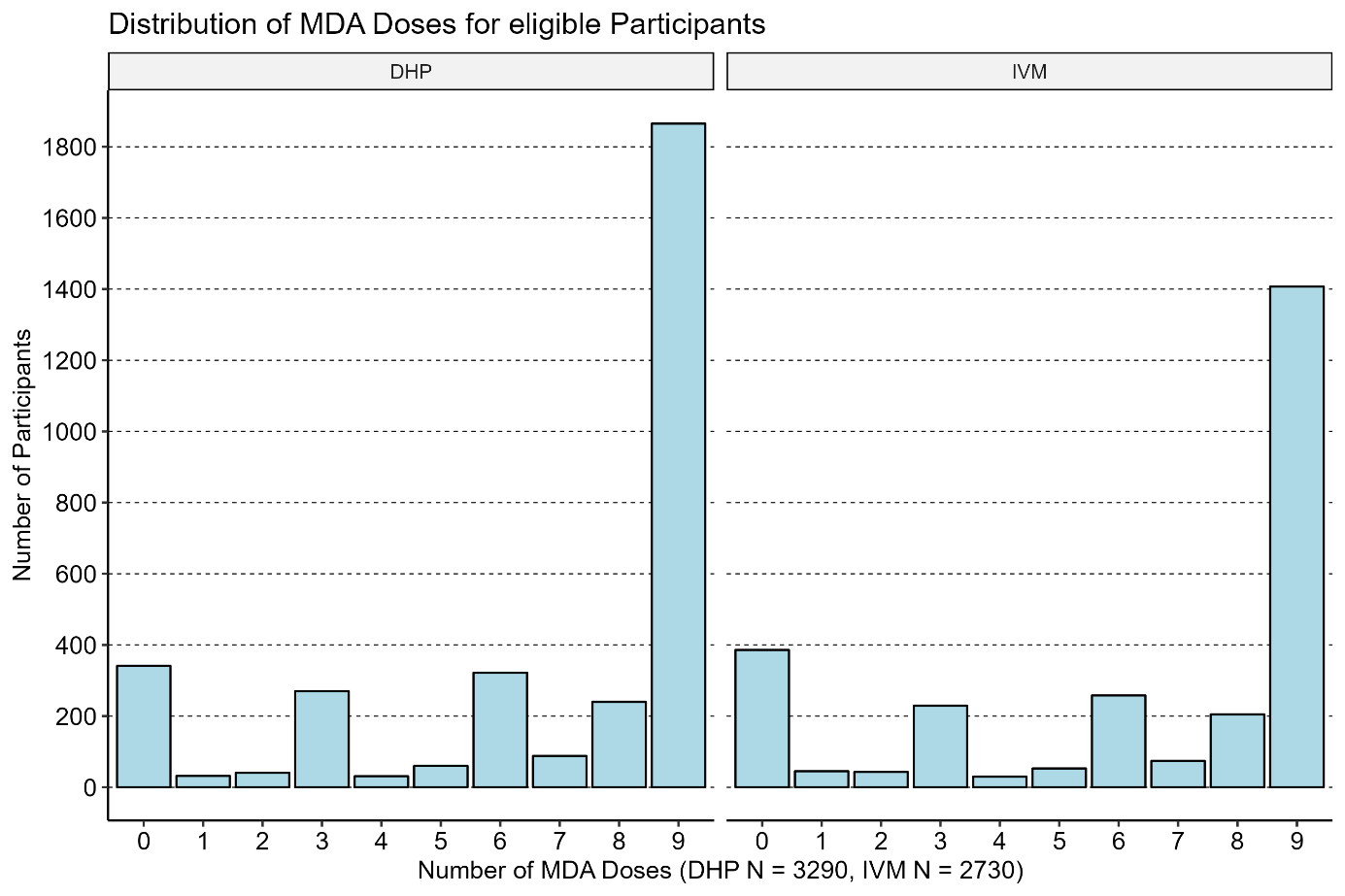


**Supplementary Figure 1.** Overall distribution of the MDA dose uptake by number of MDA doses received for IVM or DHP respectively for all eligible participants with complete data for analysis out of the total population in the intervention arm (IVM N = 2730/5036, DHP N = 3290/5036)


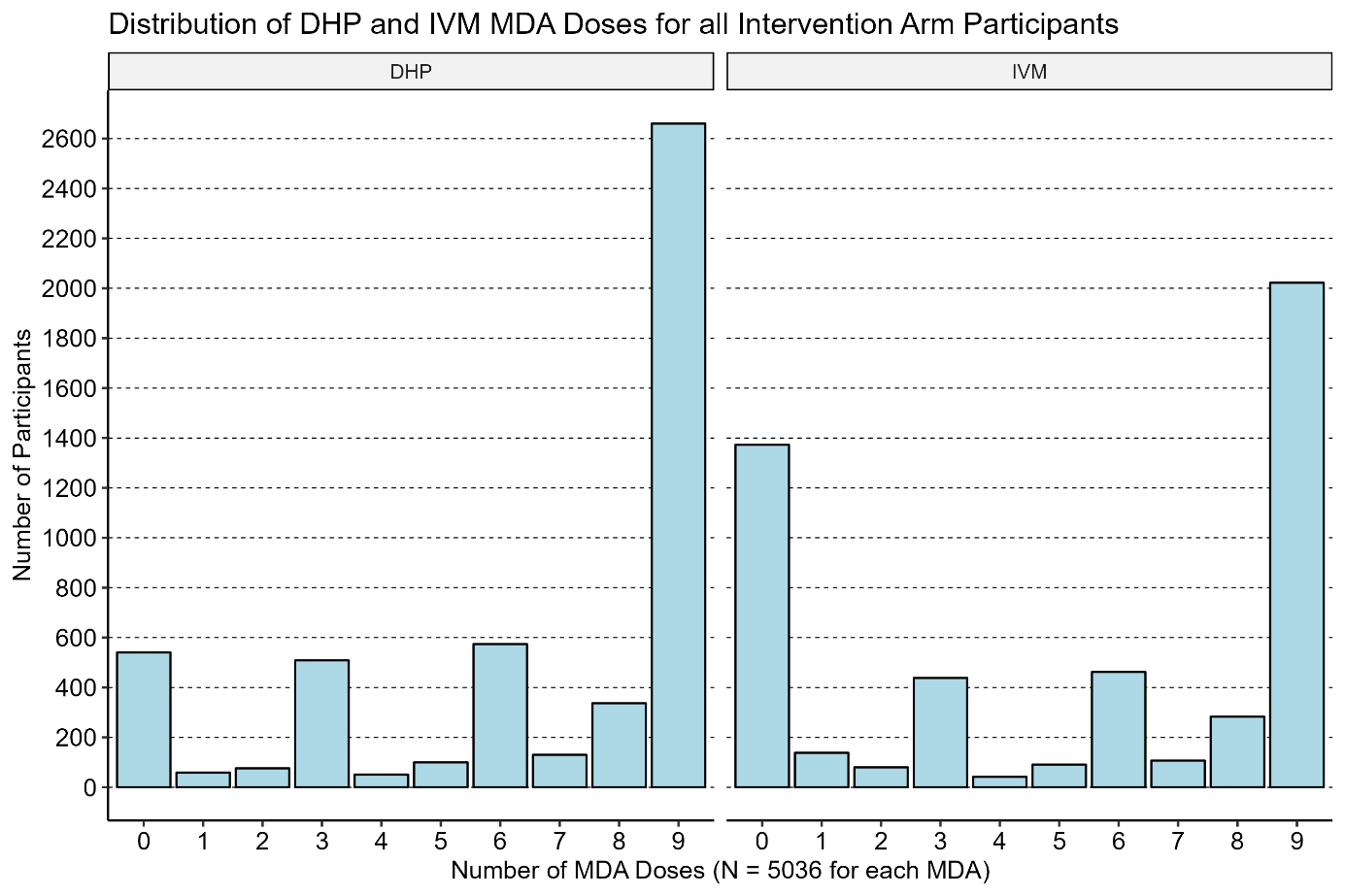


**Supplementary Figure 2.** Overall number of doses received for both DHP and IVM MDA for eligible participants including individuals with missing demographic data (N = 5036)
